# Takotsubo Syndrome in the Emergency Room — Diagnostic Challenges and Suggested Algorithm

**DOI:** 10.31083/j.rcm2304131

**Published:** 2022-04-08

**Authors:** Gassan Moady, Gal Rubinstein, Loai Mobarki, Shaul Atar

**Affiliations:** ^1^Department of Cardiology, Galilee Medical Center, 2210001 Nahariya, Israel; ^2^Azrieli Faculty of Medicine, Bar Ilan University, 1311502 Safed, Israel

**Keywords:** takotsubo syndrome, acute coronary syndrome, echocardiography, point-of-care focused cardiac ultrasound

## Abstract

Takotsubo syndrome is an important condition to consider among patients with acute chest pain in the emergency room. It often mimics acute coronary syndrome since chest pain and ECG changes are key features in both conditions. The hallmark of takotsubo syndrome is transient left ventricular dysfunction (characterized by apical ballooning) followed by complete echocardiographic recovery in most cases. Although most patients exhibit a benign course, lethal complications may occur. The use of hand-held point-of-care focused cardiac ultrasound may be helpful for early identification of takotsubo syndrome and distinguishing it from acute coronary syndrome and other cardiovascular emergencies. Emergency room physicians should be familiar with typical and atypical presentations of takotsubo syndrome and its key electrocardiographic changes. The approach in the emergency room should be based on a combination the clinical presentation, ECG, and handheld echocardiography device findings, rather than a single electrocardiographic algorithm.

## 1. Introduction

Takotsubo syndrome (TTS), also known as ‘broken heart syndrome’, ‘apical ballooning’, or ‘stress-induced cardiomyopathy’ is a type of acute reversible left ventricular dysfunction that usually occurs in elderly women following mental or physical stress [1]. The hallmark of TTS is transient left ventricular dysfunction (characterized by apical ballooning) followed by complete echocardiographic recovery in most cases. Although most patients exhibit a benign course, lethal complications may occur. The syndrome shares several features with acute coronary syndrome (ACS): chest pain, ECG changes, elevated cardiac biomarkers, and wall motion abnormality [2, 3]. The finding of apical ballooning and basal hyperkinesia matches most cases of TTS known as apical TTS. Basal, focal, and mid ventricular variants have also been reported, and account for 20% of TTS cases [4, 5, 6]. TTS should be always considered in the differential diagnosis of patients with chest pain, and physicians in the emergency room (ER) should be familiar with the clinical presentation and the required workup of this syndrome.

## 2. Background 

For the diagnosis of TTS, the modified Mayo Clinic Criteria were developed, and all four criteria are required for correct diagnosis as follows [2]:

(1) Transient left ventricle (LV) dysfunction extended beyond a single coronary artery territory.

(2) Absence of coronary artery obstruction in angiography.

(3) New ECG abnormality (ST elevation or T wave inversion) or elevated cardiac biomarkers.

(4) Absence of myocarditis or pheochromacytoma.

Later, the international Takotsubo Registry criteria (interTAK criteria) were developed to improve identification and stratification of TTS and do not consider the presence of significant coronary artery stenosis an exclusion for the diagnosis [7]. It has been estimated that about 2% of patients presenting to the ER with suspected ACS have a final diagnosis of TTS, but the incidence is probably higher among elderly women [8]. Recently, the incidence of TTS was shown to be as high as 4.6% among critically ill patients in the intensive care unit [9].

## 3. Pathophysiology

Catecholamines probably play a central role in the development of TTS by mediating various processes of epicardial coronary spasm, microvascular dysfunction and direct myocyte injury [10, 11, 12]. Clues for the essential role of catecholamines in TTS pathogenesis include high plasma levels in the affected patients, and the induction of TTS-like disease following epinephrine or norepinephrin administration [13, 14]. During catecholamine surge, epinephrine triggers β2-Adreoreceptor in cardiac tissue to switch from Gs to Gi coupling resulting in acute cardiac apical depression and ballooning [15]. Of note, biomarkers of myonecrosis such as troponin are mildly elevated in TTS except for rare severe cases, while natriuretic peptide, a marker of increased cardiac wall stress, is typically highly elevated [16]. A genetic predisposition based on polymorphism in G protein-coupled receptor kinase 5, estrogen receptors, a1 and b1-adrenergic receptor have been suggested to play a role, but the susceptibility of these genes with familial TTS yielded conflicting evidence [17].

## 4. Clinical Presentation

The typical patient with TTS is a post-menopausal female with complaints of chest pain following a stressful event, with or without dyspnea and other signs of heart failure [18]. Notably, patients with atypical TTS are usually younger and more often have neurological comorbidities compared to those with typical TTS [4, 19]. Physical or mental stressors may precede the onset of chest pain, with possible chronobiological patterns of peak occurrence in the morning and afternoon hours when stressful events are more common [20, 21]. Common precipitating factors include stressful argument, grief, public speaking, major surgery, and natural disasters. However, the absence of emotional or physical trigger does not exclude TTS. Despite detailed history taking, it has been reported that about one third of the cases lack such an obvious stressor [18]. In the study by Templin *et al*. [18] about 27% of patients with TTS had a history of neurological disease, and about 42% had a diagnosis of psychiatric illness. Chest pain and dyspnea are the presenting symptoms in about 90% of the cases. Other less common symptoms are nausea, vomiting, palpitations, headache, weakness, epigastric pain, and syncope [22]. Based on history taking, no single symptom is sufficiently specific to differentiate TTS from ACS [23]. In addition, typical and atypical variants of TTS have the same clinical presentation. Physicians in the ER should be familiar with less common presentations of TTS such as cerebrovascular accident (CVA) and ‘torsade de pointes’ secondary to QT segment prolongation [24, 25, 26, 27]. Of note, CVA in the context of TTS may be the trigger preceding the clinical onset or a consequence of the disease during the early or late period of the syndrome secondary to thromboembolic events [18]. High index of suspicion is needed in every postmenopausal patient presenting with chest pain without evidence for myocardial infarction. Most patients are hemodynamically stable and exhibit a benign course with no complications and with complete echocardiographic recovery overtime. Progressive fulminant course with cardiogenic shock or intractable pulmonary edema may occur in rare cases [28]. Based on data from the interTAK registry, about 20% of patients had a combined endpoint of in-hospital complications [18]. Some of the severe complications are ventricular arrythmia, ventricular thrombus, and ventricular rupture. Overall, the In-hospital complications and outcomes are similar between typical and atypical variants of TTS [4].

## 5. ECG Changes 

**Fig. 1.**
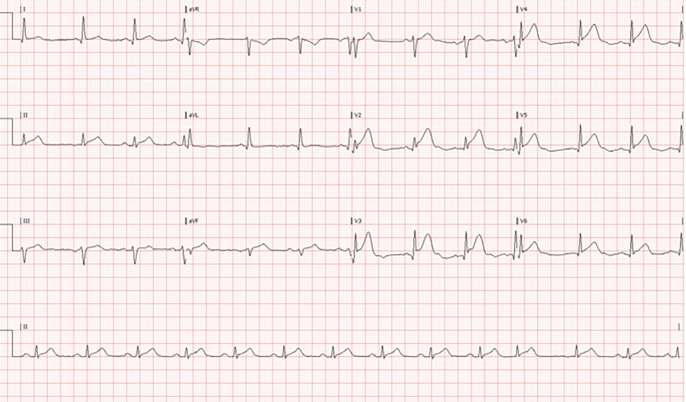
**Twelve-lead ECG of patient presented to the ER with chest pain**. Twelve-lead ECG of a 70 -year-old female who admitted the ER with acute chest pain following an argument with her daughter. The pattern of ST elevation in the precordial and inferior leads without reciprocal ST depression raised the possibility of TTS. Immediate point-of-care cardiac ultrasound (POCUS) showed apical ballooning and basal hypercontractility (displayed later), consistent with TTS.

According to the current guidelines, it is recommended to obtain twelve-lead ECG for patients with chest pain within 10 minutes after first medical contact and to have it immediately interpreted by an experienced physician [29]. The hallmark of ECG changes in patients with TTS is ST segment elevation in the precordial leads, predominantly in V2–V3, with no reciprocal changes. When interpreted by expert physician, ECG on admission has a high specificity and positive predictive value for TTS diagnosis and differentiating it from anterior myocardial infarction [30]. When comparing ST-segment elevation myocardial infarction (STEMI) to TTS, ST segment elevation in (-aVR) has a specificity of 95% and positive predictive value of 91% for TTS (*p *< 0.001). The specificity is higher when ST elevation in (-aVR) is associated with ST elevation in the inferior leads (98%) or in the anteroseptal leads (100%) [30]. On the other hand, reciprocal ST segment depression is more characteristic of STEMI. Ogura *et al*. [31] reported several ECG changes that may distinguish TTS from STEMI including the absence of reciprocal ST depression, the absence of abnormal Q waves, and the finding of sum of ST elevation in V4–V6 more than in V1–V3. When examining the evolution of ECG changes during TTS, ST segment elevation in the precordial leads develops immediately or within few hours from symptom onset. Within 1–3 days, T wave inversion may be observed after ST segment resolution [32]. Finally, after several weeks or few months, the inverted T waves may become deeper or normalize. While Q waves are common following non-perfused myocardial infarction, they are less common in TTS, and when observed, they are often transient [30]. Prolonged QTc interval is a common finding in both acute ischemia and TTS, and it is significantly longer in TTS compared to ACS [18]. We recommend that ER physicians should know the basic ECG changes in TTS for decision making rather than the use of ECG-based algorithm. The knowledge of the abovementioned changes combined with clinical presentation, echocardiography and biomarkers is more practical for ER scenario. Fig. 1 shows an ECG of 70-year-old female with TTS who was managed by ER physician.

## 6. Cardiac Biomarkers

Several cardiac biomarkers, inflammatory proteins and various ratios between them were studied to assist clinicians in diagnosing patients with TTS and to better distinguish them from patients with ACS. Of those, the most studied are Troponin (I and T), brain natriuretic peptide (BNP) and its inactive byproduct: N-terminal-pro hormone BNP (NT-proBNP), Creatine Kinase-MB (CK-MB) and Myoglobin. In patients with TTS, myonecrosis biomarkers such as troponin, CK-MB and myoglobin are elevated by a lower scale compared to ACS, due to significantly less tissue injury [33]. Several studies evaluating the role of troponin in distinguishing TTS and acute myocardial infarction (AMI) found that mean peak troponin (T and I) level is significantly lower in patients with TTS compared to patients with STEMI [33, 34, 35]. However, it should be noted that biomarker levels in both ACS and TTS may be not elevated on admission to the ER, particularly if blood samples are withdrawn early following symptoms onset. The origin of BNP secretion is primarily by the ventricles in response to stretch force. Dagrenat *et al*. [36] compared 314 patients with TTS to 452 patients with STEMI and 334 patients with non-ST elevation myocardial infarction (NSTEMI). They found that patients diagnosed with TTS had significantly higher BNP levels on admission, at peak and discharge compared to patients with STEMI and NSTEMI. The most studied and accurate biomarker ratio, to date, is the BNP/Troponin ratio that was to be significantly higher in TTS than in patients with STEMI or NSTEMI [37, 38]. A study by Randhawa *et al*. [39] revealed that a BNP/Troponin T ratio >1272 upon admission has 95% specificity for TTS when compared to AMI. Furthermore, BNP/Troponin I ratio >329 on admission distinguished TTS from NSTEMI, while better differentiation was obtained using BNP/Troponin I ratio at peak [37, 38, 39]. In addition to its immediate diagnostic value, NT-proBNP may also be utilized as a prognostic predictor to predict 30-day major adverse cardiac event (all cause death, cardiogenic shock or pulmonary edema), and long-term outcomes [40]. A few other biomarker ratios were proposed to assist clinicians in distinguishing between TTS and ACS. Troponin T/CK-MB was reported to be significantly higher in patients with TTS than in those with STEMI or NSTEMI [41]. Randhawa *et al*. [39] reported that a BNP/CK-MB ratio ≥29.9 distinguished TTS from AMI with 95% specificity and 50% sensitivity. Furthermore, NT-proBNP/Myoglobin ratio of 3.8 and 14 were suggested to properly distinguish patients with TTS than in those with STEMI (sensitivity: 89%, specificity: 90%) and NSTEMI (sensitivity: 65%, specificity: 90%), respectively [39]. While the exact cut off values for different laboratory parameters are varied and differ from trial to trial, the laboratory trend in TTS is clear: the rise in Troponin, CK-MB and Myoglobin is disproportionally low whereas BNP is dramatically elevated. Since atypical variants of TTS involve less extent of the myocardium, patients tend to have higher left ventricular ejection fraction and lower NT-proBNP levels compared to typical TTS [4].

## 7. Role of Point-of-Care Focused Cardiac Ultrasound

**Fig. 2.**
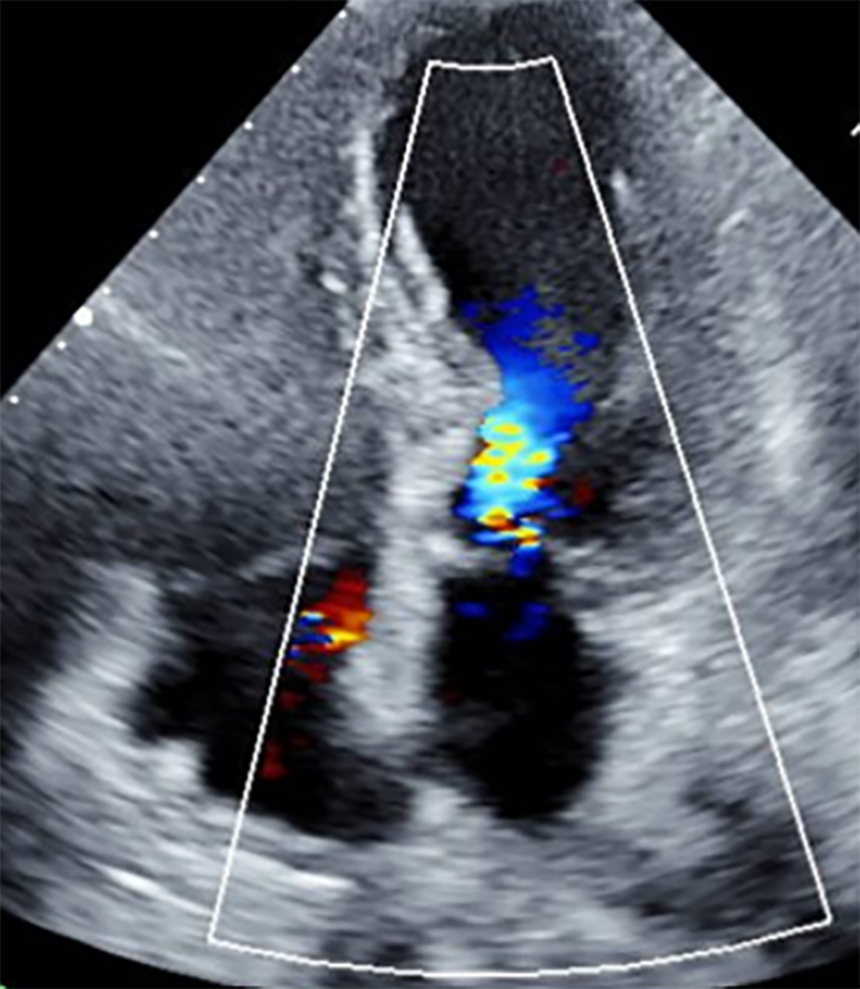
**Apical four chamber view using handheld POCUS**. Apical four-chamber view of the patient performed by ER physician showed typical apical ballooning and LVOTO secondary to basal hypercontractility.

The use of hand-held point-of-care ultrasound (POCUS) has become a key component in rapid triage of patients with acute chest pain. This is particularly important in the ER setting due to the scarce resource of the traditional big machines along with simplicity and availability of this useful miniaturized modality. The use of POCUS provides a quick evaluation of patients’ cardiovascular status and helps in guiding the management by the primary care givers in the ER. Several emergent conditions may be identified using this modality including cardiac tamponade, myocardial infarction, and pulmonary embolism [42, 43, 44]. Early ER sonography using POCUS was also shown to be useful in TTS diagnosis [45, 46]. Educational sessions in POCUS training for ER physicians were shown to be valuable. Targeting distinct conditions such as pericardial effusion, aortic dissection, TTS, and pulmonary embolism may be more practical than performing a whole examination, which may detect incidental findings [47]. The use of hand-held devices becomes more valuable in the era of the novel coronavirus disease 2019 (COVID-19) since conventional imaging modalities such as transthoracic echocardiography and invasive coronary angiography should be restricted to minimize physician-patient contact. The use of handheld POCUS is highly appropriate for this purpose. These devices offer a portable and inexpensive modality that along with physical examination may provide a comprehensive evaluation of the cardiovascular system. Although the handheld device does not provide the high diagnostic power and some modalities such as three-dimensional imaging as the conventional machine, it constitutes a useful device in the ER scenario. Many schools have adopted the training in the use of these devices by medical students as part of the curriculum. Fig. 2 shows an apical four chamber view obtained using handheld POCUS in the ER.

**Fig. 3.**
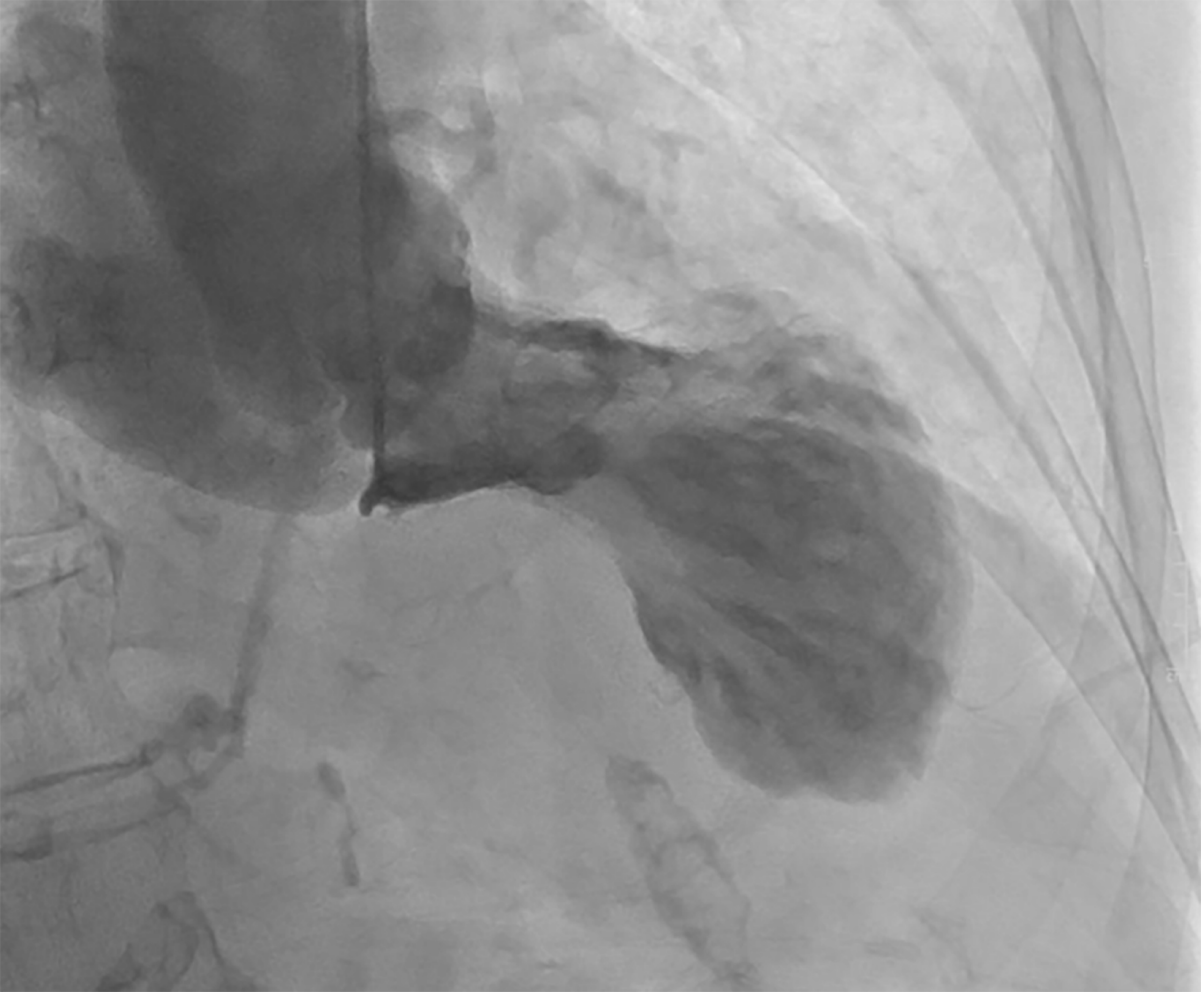
**Ventriculogram of the same patient during cardiac catheterization**. Ventriculogram of the same patient who was suspected for TTS based on POCUS in the ER. Invasive coronary angiography was performed later and revealed patent coronary arteries without obstruction. Left ventriculogram showed typical apical ballooning, confirming the diagnosis of TTS.

Diagnosis of TTS was assumed based on the clinical presentation, ECG, and echocardiographic appearance. Patient was not treated with antiplatelet therapy and was directly admitted to cardiac unit for further management. The use of handheld echo devices if performed by experienced physician may support the diagnosis of TTS in the apical four-chamber view. The same patient was sent to cardiac catheterization, and her ventriculogram is provided in Fig. 3.

## 8. Diagnostic Workup and Suggested Algorithm

**Fig. 4.**
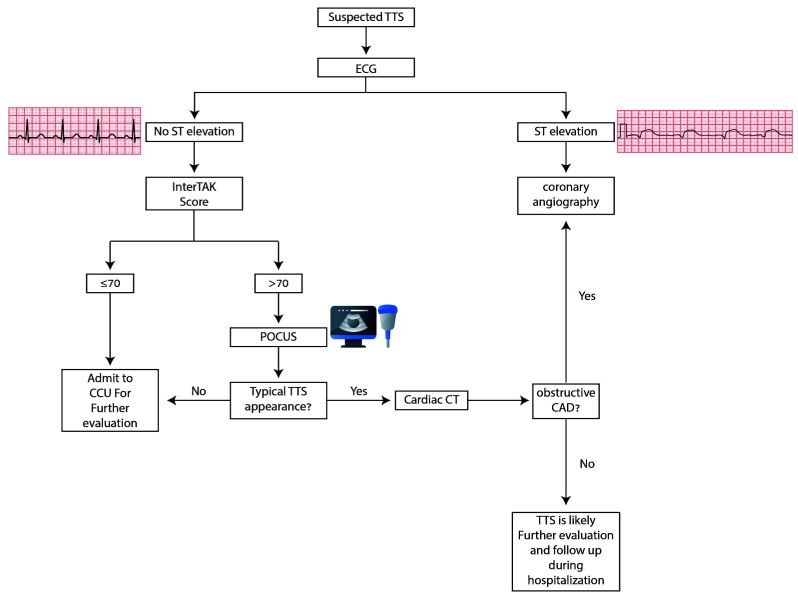
**Diagnostic algorithm**. In stable patients, ECG within 10 minutes is recommended in patients with chest pain. When ST elevation is present, invasive coronary angiography should be performed to rule out obstructive disease. In patients without ST elevation, the interTAK score may help in patient triage. A score >70 should encourage POCUS and subsequent cardiac CT when the findings are consistent with TTS. If the probability for TTS is low (<70), and in the cases that POCUS does not support TTS diagnosis, admitting to the cardiac care unit (CCU) for further evaluation is recommended. *Obstructive CAD may be present in TTS, however it should not be in a distribution that explains the observed wall motion abnormalities.

Other than acute coronary syndrome, several emergent conditions such as aortic dissection and pulmonary embolism manifest with chest pain similar to TTS and should be ruled out in the ER. The use of triple rule-out computed tomography (TROCT) was investigated in patients with acute chest pain in several studies, including in the ER context [48]. This modality may be beneficial in certain cases due to the ability to identify both cardiac and non-cardiac conditions in equivocal cases of chest pain. TRO-CT simultaneously examines the coronary arteries, thoracic aorta, and pulmonary artery, and may detect coronary lesions, aortic dissection, and pulmonary embolism respectively [49]. In one study, TRO-CT detected significant noncoronary diagnosis in about 9% of patients admitted the ER with chest pain, including findings that would not be identified in cardiac CT modality [50]. It should be noted, however, that the use of TRO-CT is limited in some centers and requires multidisciplinary team radiologists 24/7 availability. Although TTS generally has more benign course than AMI, rapid diagnosis is essential to avoid unnecessary, and potentially harmful, treatment. The interTAK score was developed to predict the probability of the diagnosis of TTS based on seven variables and each was assigned a score value: female sex 25, emotional trigger 24, physical trigger 13, absent of ST depression (except in aVR) 12, psychiatric disorder 11, neurologic disorder 9, and QTc prolongation 6 points [51]. We recommend the use of the interTAK score to decide which patient needs further evaluation by handheld POCUS in the ER. Echocardiography is essential for identification of potential mechanical complications such as LVOTO, acute mitral regurgitation, and thrombus formation [52]. The use of invasive coronary angiography as the initial diagnostic tool should be restricted to patients with ST-segment elevation (even in the presence of apical hypokinesis) to exclude left anterior descending artery occlusion. In other cases, cardiac CT is appropriate for demonstrating the coronary arteries and for definitive diagnosis of TTS. The following algorithm (Fig. 4) summarizes our suggested diagnostic approach in the ER. The use of this simple algorithm in the ER is feasible since it depends on clinical judgment and the interTAK score.

## 9. Treatment 

In most cases, treatment of TTS is conservative with focus on mental and physical stress relieve. When LV dysfunction is present, treatment with beta-blockers and Angiotensin-converting enzyme (ACE) inhibitors is reasonable. Despite the lack of randomized controlled trials, the use of ACE inhibitors and beta-blockers could be associated with improved survival by reducing the risk of malignant arrythmia, cardiac rupture, and cardiogenic shock [53, 54]. Caution is needed when there is left ventricular outflow tract obstruction (LVOTO). In such cases, inotropic agents are contraindicated, and beta-blockers may be beneficial in reducing basal hypercontractility and relieving the obstruction [53]. Once TTS diagnosis is definitive, antiplatelet therapy is not recommended and may be associated with increased mortality [55, 56, 57]. Despite the role of catecholamine in the pathogenesis of TTS, there is no consensus about the role of beta-blockers in reducing TTS recurrence [58]. It should be emphasized that the overall prognosis of TTS patients is comparable to that of ACS [18, 59]. Physicians in the ER should know how to distinguish TTS among a myriad of patients presenting with acute chest pain. Rapid diagnosis is essential in order to avoid unfavorable outcomes.

## 10. Considerations during the COVID-19 Era 

The novel coronavirus disease 2019 is associated with several cardiovascular manifestations including myocardial injury, myocarditis, arrhythmia, acute coronary syndrome, and pulmonary embolism [60, 61, 62, 63]. Rare cases of TTS have been reported as a complication of the acute infection and secondary to the overwhelming stress accompanying this outbreak [64, 65, 66]. Diagnosis in patients tested positive for COVID-19 is challenging since conventional imaging modalities such as transthoracic echocardiography and invasive coronary angiography should be restricted to minimize physician-patient contact [67]. The use of handheld POCUS is of paramount significance particularly during the current pandemic. One of the major impacts of the current pandemic are its psychological and social effects, mainly among elderly. The social deprivation, which became a direct consequence of COVID-19, may jeopardize patient adherence to therapy, routine medical check-up and follow-up visits, which in turn aggravates depression and anxiety, creating a vicious cycle. The issue whether TTS incidence was affected by COVID-19 burden was addressed in several studies with conflicting results [68, 69, 70]. Overall, there appears to be an association between TTS incidence and COVID-19 since this syndrome is mainly mediated by stress-related pathways [16].

## 11. Conclusions

Physicians in the ER should be familiar with various clinical manifestations and major electrocardiographic changes of TTS. One of the important available diagnostic tools to distinguish it from other cardiovascular conditions is the handheld POCUS device. Training in the use of these devices has become a part of the curriculum for medical students. The use of a simple algorithm in the ER may facilitate the triage of patients with suspected TTS and avoid unnecessary treatment.
